# In-Situ X-ray Tomography Observation of Structure Evolution in 1,3,5-Triamino-2,4,6-Trinitrobenzene Based Polymer Bonded Explosive (TATB-PBX) under Thermo-Mechanical Loading

**DOI:** 10.3390/ma11050732

**Published:** 2018-05-04

**Authors:** Zeng-Nian Yuan, Hua Chen, Jing-Ming Li, Bin Dai, Wei-Bin Zhang

**Affiliations:** 1 Institute of Chemical Materials, China Academy of Engineering Physics, Mianyang 621900, China; znyuan@foxmail.com (Z.-N.Y.); daibin@caep.cn (B.D.); weibinzhang@caep.cn (W.-B.Z.); 2 Graduate School of China Academy of Engineering Physics, China Academy of Engineering Physics, Mianyang 621900, China

**Keywords:** in-situ X-ray computed tomography, thermal-mechanical loading, polymer bonded explosives, mesoscale characterization, structure evolution

## Abstract

In order to study the fracture behavior and structure evolution of 1,3,5-Triamino-2,4,6-Trinitrobenzene (TATB)-based polymer bonded explosive in thermal-mechanical loading, in-situ studies were performed on X-ray computed tomography system using quasi-static Brazilian test. The experiment temperature was set from −20 °C to 70 °C. Three-dimensional morphology of cracks at different temperatures was obtained through digital image process. The various fracture modes were compared by scanning electron microscopy. Fracture degree and complexity were defined to quantitatively characterize the different types of fractures. Fractal dimension was used to characterize the roughness of the crack surface. The displacement field of particles in polymer bonded explosive (PBX) was used to analyze the interior structure evolution during the process of thermal-mechanical loading. It was found that the brittleness of PBX reduced, the fracture got more tortuous, and the crack surface got smoother as the temperature rose. At lower temperatures, especially lower than glass transition temperature of binders, there were slipping and shear among particles, and particles tended to displace and disperse; while at higher temperatures, especially above the glass transition temperature of binders, there was reorganization of particles and particles tended to merge, disperse, and reduce sizes, rather than displacing.

## 1. Introduction

Polymer bonded explosive (PBX) is a class of heterogeneous composite material that mainly consists of explosive crystal particles and polymer binder matrix. PBX is widely used in weapon systems because of its excellent performance. In the course of the service of the weapon systems, because of various environment temperature and mechanical actions, such as compression and tension, there could be a series of damage occurring, structure evolution, and even fracture behaviors in PBX. These damages, structure evolution, and fractures directly affect the mechanical properties, safety performance, and detonation performance of PBX [1,2]. Therefore, it is important to study the response of PBX under thermo-mechanical loading. 

Previous researches mainly studied the single-factor effect on PBX. When considering the mechanical loading, the mechanical properties of PBX were widely studied [3,4]. The deformation [5], creep [6], cohesive [7], and fracture [8] behavior of PBX under mechanical loading were investigated in the digital image correlation (DIC) method. The micro-mechanical evolution was investigated through scanning electron microscopy (SEM). It was found that there was a variety of types of damages in PBX, such as intragranular voids, crystal fractures, interfacial debonding, and deformation twinning [9,10,11]. The above detection techniques could only obtain the surface morphology, however X-ray micro-computed tomography (μCT) allows for the observation of the internal three-dimensional (3D) structures. The internal deformation of PBX simulant in compression was analyzed in detail by digital volume correlation (DVC) of in-situ μCT [12]. 

While in the terms of thermal loading effects, state and phase change of both explosives [13] and binders [14] at a wide range of temperatures were studied. The mesoscale structure evolution of PBX during heating was analyzed by ultra-small angle X-ray scattering (USAXS) and μCT [15]. However, mechanical properties and structure evolution of PBX under thermal-mechanical coupling loading still need to be investigated deeply. Because of the technical difficulties of experiments, most of the researches on properties of PBX in thermal-mechanical loading were simulation calculations [16,17]. In respect of experiments, Willamson and others comprehensively studied temperature-time response of an cyclotetramethylenetetranitramine (HMX)-based PBX at a wide temperature range [18]. It was found that the failure strain of PBX is non-sensitive to temperature, so the modules of PBX at different temperatures have a liner relation with failure stress. Other researchers studied the mechanical [19,20,21] and fracture [22] behavior of PBX under different temperature and loading conditions from different perspectives. The previous researches were mainly focus on the mechanical properties of PBX at high temperatures, while the structure evolution of PBX at both low and high temperatures still needs more investigation. Especially, most of the researches were about HMX-based PBX, while few researches studied 2,4,6-triamino-1,3,5-trinitrobenzene (TATB)-based PBX. Furthermore, quantitative characterization techniques should be built up to describe and analyze the observation result of fracture and structure evolution in mathematical language. 

In this paper, in-situ Brazilian test with an improved arc loading head was conducted on aμCT apparatus in order to investigate the interior structure evolution of a TATB-PBX. The test was under quasi-static loading and five different temperatures, ranged from −20 °C to 70 °C. Three-dimensional morphology of cracks was investigated by digital image process. Fracture degree and complexity were defined and used to quantitatively characterize the crack properties. Fractal dimension was used to characterize the roughness of the crack surface. The test samples were also investigated by SEM, and the results of different kinds of detection and analysis were compared. Slice images of μCT were also analyzed by the displacement of particles, and the displacement field of the interior structure of PBX was analyzed. 

## 2. Materials and Methods 

**Figure 1:**
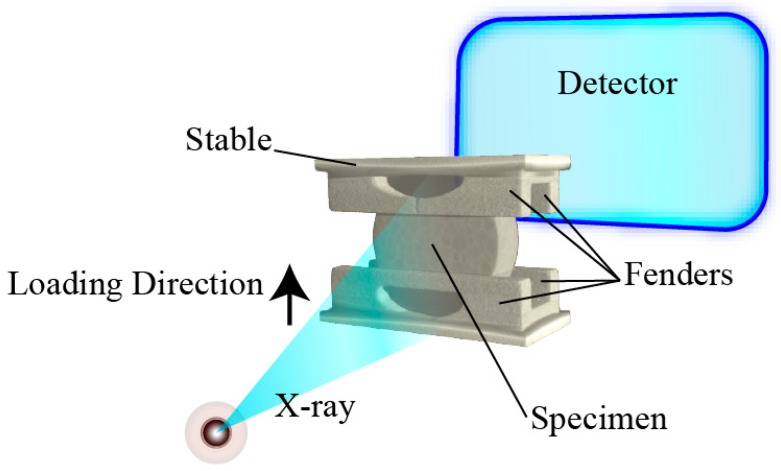
The loading method diagram.

The experimental specimen is a kind of TATB-based PBX. The main components are TATB as explosive and F2314 fluororubber as binder. TATB and F2314 are firstly made into molding powders, which are particles with some TATB powder crystals that are wrapped in an F2314 binder. The mass percentage of TATB in this PBX is higher than 90%. The specimen was made into a disk with diameter of 10 mm, and a thickness of 3 mm. The experiment took the way of Brazilian disc quasi-static displacement loading, the loading speed was 0.1 mm/min. As a method of indirect tension, Brazilian disc test is widely used to study the damage behavior of brittle materials. Awaji and Sato [23] improved the loading head in diameter disc compression to reduce the stress concentration at the head and analyzed the stress distribution under this type of loading. Pang [24,25] and others found that when the radius of the arc loading head is 1.35 times of the radius of the specimen, the test result is the closest to that of the direct tensile test. In this experiment, the radius of the arc loading head was designed in this method. Because of the small size of the specimen, and in order to ensure the stability of the specimen, a group of fenders were used in experiment. The test loading diagram was shown in Figure 1. 

The experiment was carried out with the same scan parameters in every group of tests in order to ensure that the scan results are consistent and comparable. The scan voltage was 60 kV, scan current was 150 mA, exposure time was 0.4 s, and with an image merging number of 5. In this condition, the spatial resolution of this experiment was 21.182 μm/voxel. The loading device was an in-situ CT material testing machine, Deben Microtest CT5000-TEC (Deben UK Ltd, London, UK). In every group of test, the specimen was firstly scanned at room temperature without any loading for structure information of original state. Then, the test temperature, −20 °C, 0 °C, room temperature (22 °C), 55 °C (the glass transition temperature of F2314 is around 50 °C), or 70 °C was set, and we waited for 30 min in order to make the specimen temperature stable. Then, the specimen was loaded to fracture, and was scanned under the fracture state. Replicated tests were taken in order to ensure the repeatability and accuracy of the experiment. The specimen was also loaded to 75% fracture extension at different temperatures, which was obtained in the previous tests, and was scanned to get the structure information of intermediate state of loading at different temperatures. After in-situ CT tests, some of the specimens were also detected by SEM (CamScan Apollo300, CamScan, Cambridgeshire, UK) for more information of more micro-scale structure to compare with the results of CT detection. 

## 3. Results

### 3.1. Stress-Extension Curves at Different Temperatures

Using Deben Microtest CT5000-TEC device, an extension-force curve in the test can be obtained and recorded automatically, and the stress can be derived by the following expression [10],

(1) $$\sigma_{xx}=\left(1-{\left(\frac{b}{R}\right)}^{2}\right)\frac{2P}{\pi Dt}$$

where *P* is the loading force and *D* and *t* are the specimen’s diameter and the thickness, respectively, *b* is the contact half-width of the anvils and *R* is the radius of the specimen. This *b*/*R* ratio should be measured for the specimen in experiments. This ratio is recommended to be greater than 0.27, since this has been found the failure is to be purely tensile [10]. In this paper, all of the *b*/*R* ratios in every group of tests are around 0.28 to 0.30.

**Figure 2:**
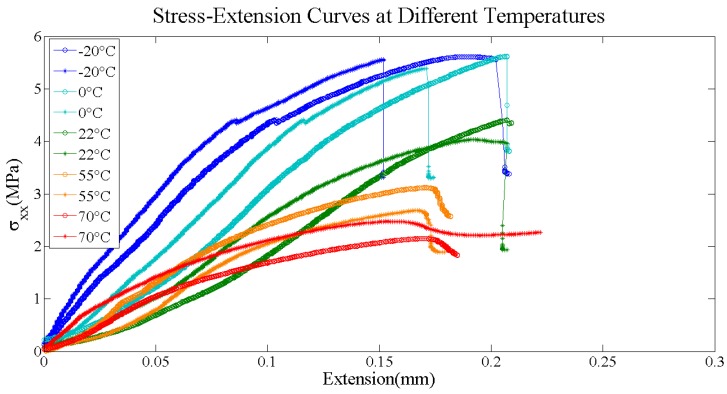
Stress-extension curves at different temperatures, at each temperature there is a repeat group.

Through Formula (1), stress-extension curves in every test can be obtained. The curves at different temperature were shown in Figure 2.

As we can see from Figure 2, all of the failure extensions are around 0.15 to 0.20 mm, the difference at the same temperature tests basically equals to the difference among different temperature groups. Therefore, the failure extension is not sensitive to the temperature, which agrees to the previous research [18]: temperature has little effect on tensile failure strain. As the temperature decreases, the binder hardens, the PBX modulus increases, and the failure stress increases accordingly. As the temperature increases, the binder changes to high elastic state, the viscosity increases, the PBX modulus decreases, and the failure stress reduces, accordingly. In low temperature conditions, such as room temperature, 0 °C and −20 °C, the brittleness is significant, the specimen loses its carrying capability at the fracture moment; and, at 55 °C, the load of specimen decreases a little after fracture; when temperature reaches 70 °C, the sample does not lose carrying capability, but continue to load, and an obvious creep occurs.

### 3.2. CT Images and Digital Image Process

**Figure 3:**
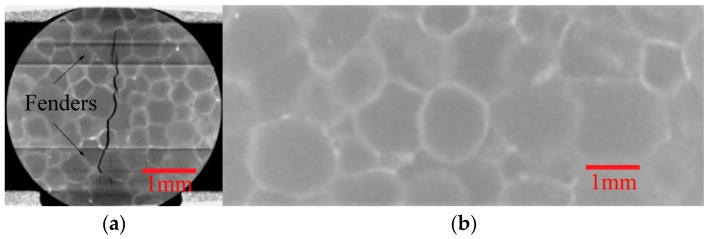
(**a**) Left one is the computed tomography (CT) slice with cracks. Because the loading head used in Brazilian test in this paper has fenders (as shown in Figure 1) in order to stabilize the specimen, two dark horizontal lines can be seen in the figure; (**b**) Right one is the CT slice without cracks.

**Figure 4:**
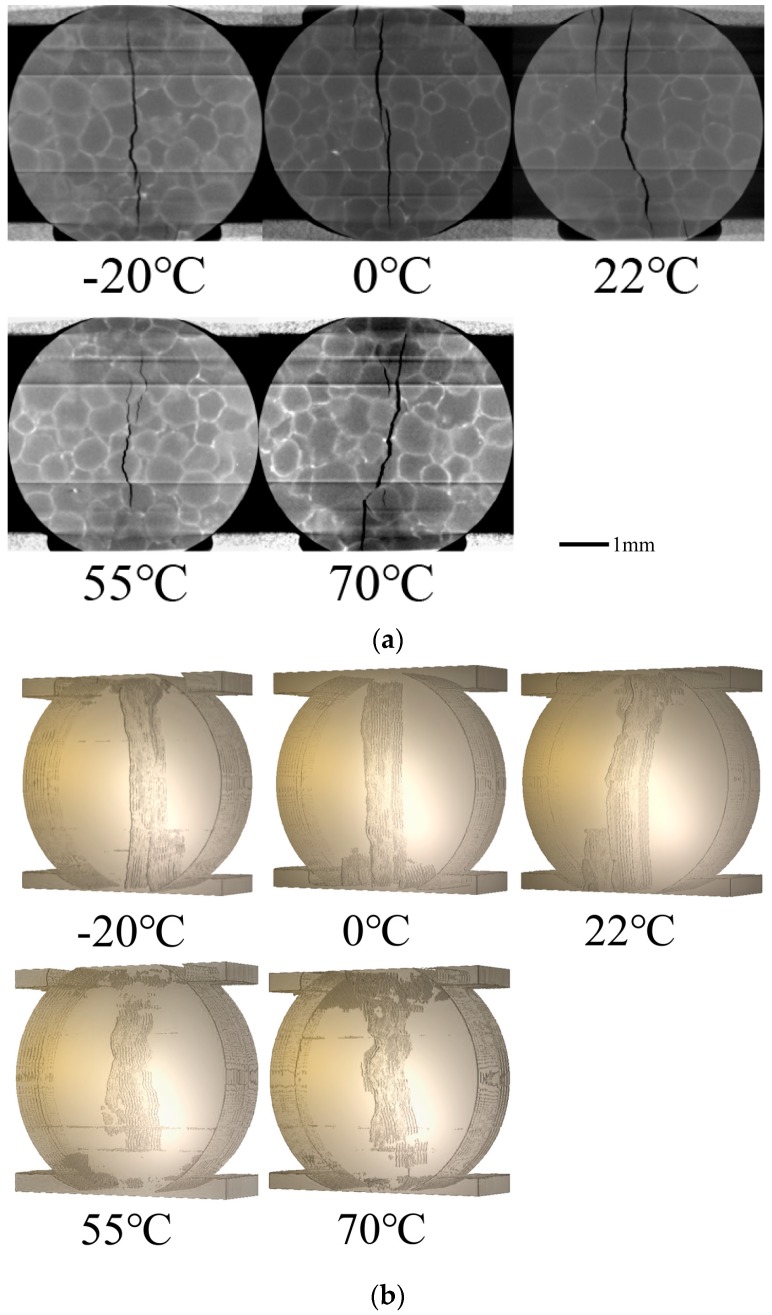
(**a**) Typical slice images of cracks at different temperatures; (**b**) Three-dimensional (3D) crack morphology at different temperatures.

A typical slice image from CT scan and image reconstruction is shown in Figure 3, (a) is the slice of fractured specimen and (b) is the slice of original one. Generally speaking, the grayscale of the image is proportional to the mass density, so different components in the specimen can be identified. However, in this kind of PBX, the mass densities of explosives (TATB molding powder) and binders (F2314) are very close. The density of TATB is 1.93 g/cm^3^ and the density of F2314 is around 2.04 g/cm^3^. Because of beam hardening and other system errors that are caused by CT, components in the specimen cannot be simply identified and segmented only by grayscale histogram. In this paper, several digital image processing methods, including morphological image processing (such as open and close operations and so on) and various segment techniques (such as Otsu segment and so on) were used to segment explosives (TATB molding powder), binders (F2314), and cracks, and to process the CT slices into binary images. The algorithms and criterion of the grayscale threshold used in segmentation are same in all of the analyses of experiments. The typical cracks at different temperatures are shown in Figure 4a. Three-dimensional crack morphologies can be obtained through reconstruction of binary images, shown in Figure 4b, and more analysis of these binary slices are in the next section.

As is shown in Figure 4, the cracks are straighter at lower temperatures, while they are more tortuous at higher temperatures. This is because, at lower temperatures, the fracture has significant brittleness; while at higher temperatures, as the binders turn into high elastic state, the viscoelasticity of the specimen enhances, so crack paths are more along the grain boundaries, then a more tortuous crack will occur.

### 3.3. Fracture Mode Comparison

**Figure 5:**
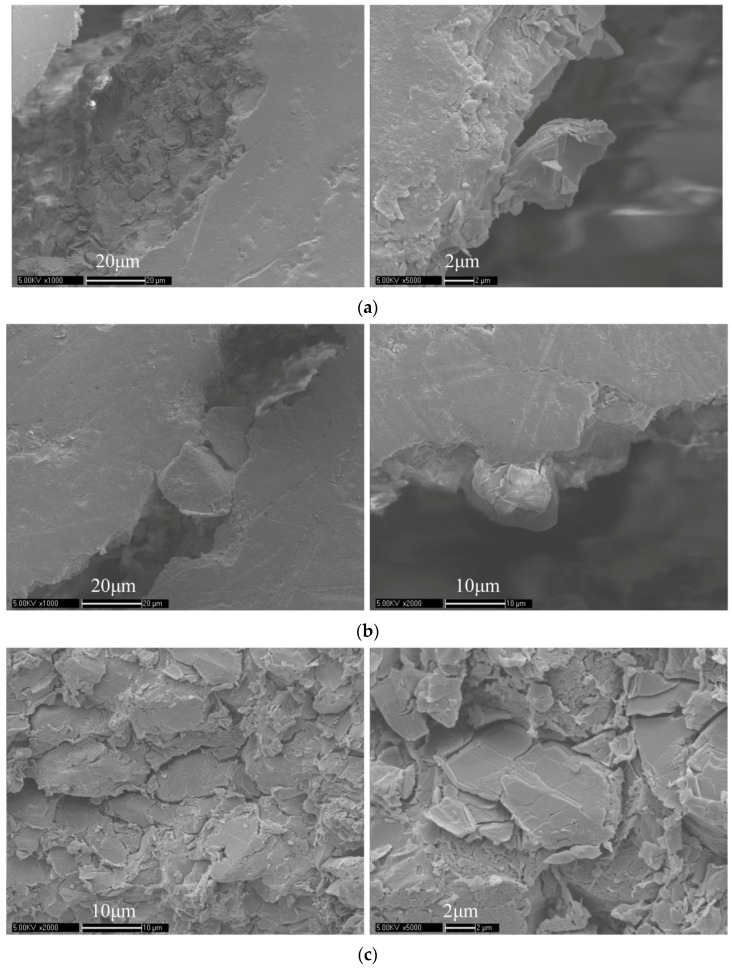

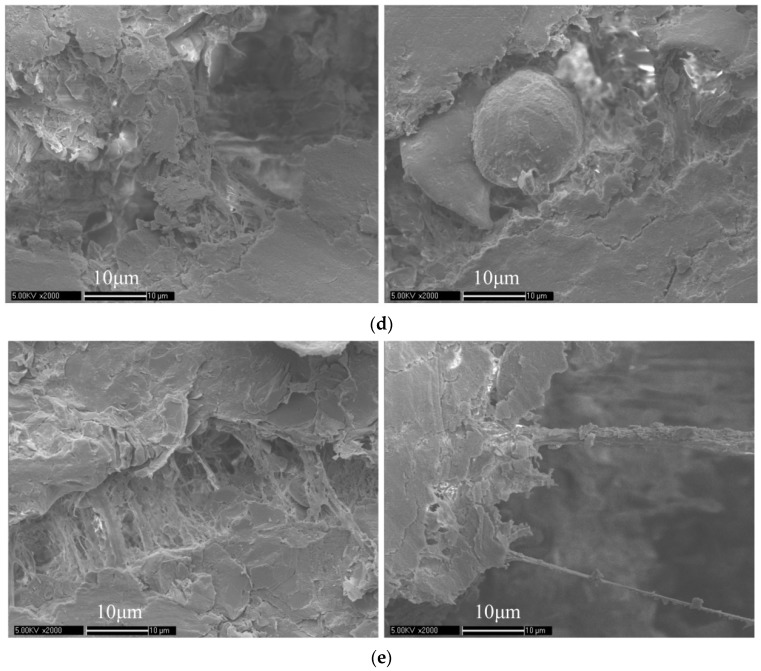
(**a**) SEM images in −20 °C; (**b**) SEM images in 0 °C; (**c**) SEM images in 22 °C; (**d**) SEM images in 55 °C; (**e**) SEM images in 70 °C.

The fractured specimens at different temperatures were also detected by SEM. The result of SEM detection proves the result of fracture fractal dimension analysis. Figure 5 shows some typical images at different temperatures, −20 °C, 0 °C, 22 °C, 55 °C, and 70 °C, respectively: 

The SEM images show the great difference of the fractures at different temperatures. Figure 5a is SEM images in −20 °C, the rough fracture surface can be clearly seen and the main fracture mode is the break of particles and crystals. Because of the angular broken particles and crystals, the fracture surface is rougher, resulting in a larger fracture fractal dimension. Figure 5b is SEM images in 0 °C, a large amount of fractured particles drop out. Figure 5c is SEM images in 22 °C. These are images of the fracture surface, which can be seen directly that it is rough. Meanwhile, not only broken particles and crystals can be seen, but there are also some broken binders around the fracture surface. Figure 5d is SEM images in 55 °C. The binders bridging the gap of particles in cracks can be seen clearly, as shown in the left image. Some large single particles drop out, a smooth surface that is covered with binders can be seen clearly, which implies a smaller fracture fractal dimension. Because the glass transition temperature of the binder is around 50 °C, in and above this temperature the fracture mode is mainly transgranular and debonding. Figure 5e is SEM images in 70 °C. There are lots of binders bridging the cracks along the crack path. The main fracture mode can be clearly and directly seen through SEM images, which agrees with the previous researches and the result of fracture fractal dimension, which will be discussed in the next section.

## 4. Discussion

### 4.1. Degree, Complexity, and Fractal Dimension of Fracture

In order to characterize the fracture morphology characteristics and to link them with mechanical behaviors, quantitative digital characterization techniques are required. In this paper, a characterizing method was defined and used to analyze the experiment results, including fracture degree and complexity, and fractal dimension of the cracks. Through this method, different types of fracture morphology can be distinguished quantitatively, and the results were also proved by SEM detection. 

Fracture degree *D* is defined as the percentage of the volume of the cracks (i.e., the void volume) in the specimen; while the fracture complexity *C* is defined as the ratio of the fracture surface area to the fracture volume. In order to non-dimensionalize this fracture complexity, the fracture surface area should be multiplied with a voxel width. As shown in Formulas (2) and (3),

(2) $$D=\frac{V_{fracture}}{V_{specimen}}$$

(3) $$C=\frac{S_{fracture}\cdot W_{voxel}}{V_{fracture}}$$

where $V_{fracture}$ is the volume of fractures, $V_{specimen}$ is the volume of specimen, $S_{fracture}$ is the area of fracture surface, and $W_{voxel}$ is the voxel width. 

Fracture degree is used to characterize the level of the fractures. A larger fracture degree describes a more serious fracture; while, the fracture complexity is used to characterize the tortuosity character of the fractures. A larger fracture complexity describes a more tortuous fracture. In porous media, tortuosity is commonly used to describe diffusion [26], which is defined as the arc-chord ratio: the ratio of the length of the curve to the distance between the ends of it. However, the real fractures are in three-dimensional, the whole volume and surface area of the fractures should be considered. Therefore, the fracture complexity is defined in this way. 

**Figure 6:**
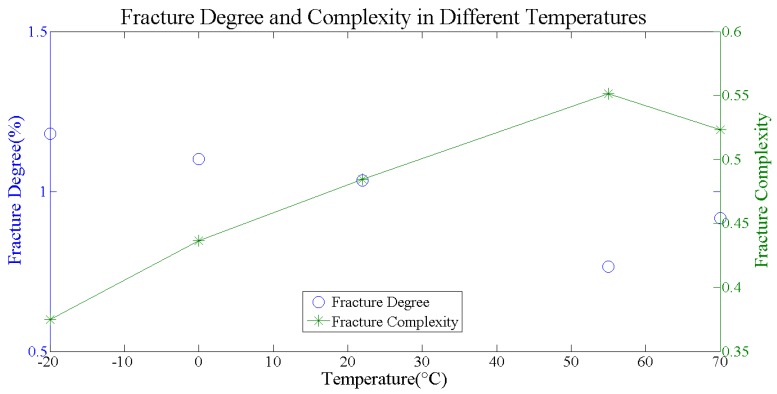
Fracture degree and complexity at different temperatures.

The fracture degree and complexity of cracks at different temperatures are shown in Figure 6. The fracture degree shows a slight fluctuation around 1% at different temperatures, while the fracture complexity shows an increasing trend with the rise of temperature. Because, when temperature rises, the brittleness of the specimen will be weakened, while the viscosity will be enhanced. Therefore, the cracks at lower temperature will be more straight, while at higher temperature, it will be more tortuous, which can be also seen from the 3D crack morphology image directly (shown in Figure 4). 

Fractal dimension of fracture is used to describe the roughness of the crack surface. Fractal geometry was firstly formed and defined in order to describe fractal features in 1983 [27]. Fractal feature is a self similarity of geometric characteristics, which means that the object always has a self-similar structure at a smaller scale. Obviously, if the fracture has a fractal feature, then the crack surface is not smooth in a micro-scale. Therefore the fractal dimension of fracture is commonly used to describe the roughness of the crack surface [28]. Previous research in brittle materials found that the fractal dimension of fracture is related to fracture toughness [29,30,31,32,33]. This can be explained as that a larger fracture fractal dimension implies a larger ratio of transgranular or trans-particle fracture, while a smaller fractal dimension implies a larger ratio of intergranular or inter-particle fracture. 

In this paper, a box-counting method was used to calculate the fractal dimension of cracks at different temperatures. The box-counting dimension is defined, as following:

(4) $$d=\lim_{\varepsilon \to 0}\left(\frac{\log N\left(\varepsilon \right)}{\log\left(1/\varepsilon \right)}\right)$$

where *d* is the fractal dimension, ε is the chosen scale, N(ε) is the volume of the crack under the scale ε. The algorithm based on box-counting dimension goes as following:

- choose a series of different sizes of cubes, $\left\{\varepsilon_{i}\right\}$;
- use these cubes to fill the cracks, count the amount of cubes used, $\left\{N\left(\varepsilon_{i}\right)\right\}$; and,
- fit $\left\{\log\left(N\left(\varepsilon_{i}\right)\right)\right\}$ and $\left\{\log\left(1/\varepsilon_{i}\right)\right\}$.

**Figure 7:**
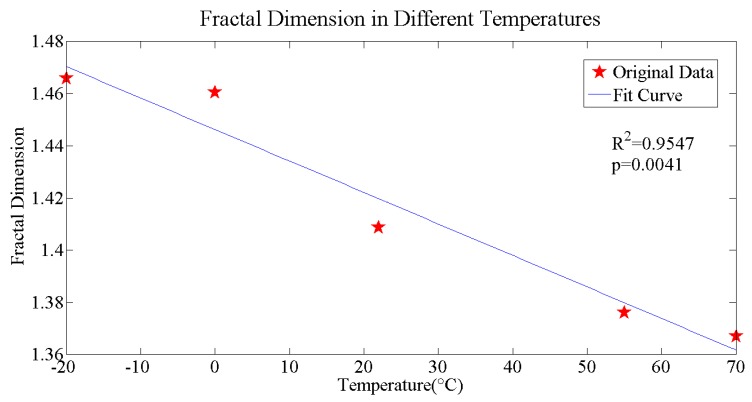
The relation of fracture fractal dimension and temperature.

If there is a strong linear correlation, then the fractal characteristic is typical and obvious, and the slope is the fractal dimension of the fracture. In this paper, all of the *R^2^* in fractal dimension fit is above 0.99, with the *p*-value being far smaller than 0.01, which means that the cracks have a typical and obvious fractal characteristic. The fracture fractal dimensions at different temperatures are shown in Figure 7. 

The fracture fractal dimension showed a decreasing trend with the rise of temperature, which is rational because at a lower temperature, especially lower than the glass transition temperature of the binder, the brittleness of binder is enhanced, so more transgranular and trans-particle fracture will occur; while at a higher temperature, especially higher than the glass transition temperature of the binder, the viscosity of the binder is enhanced so more intergranular and inter-particle fracture will occur. This result agrees with the previous research on TATB-PBX by atomic force microscopy (AFM) [34]. This phenomenon can also be seen directly through SEM images, which was discussed in the previous section. 

### 4.2. Interior Displacement Field of PBX

Through calculating the morphological connectivity of the binary images that were obtained from CT slices, the “center of pixel mass” of each particle can be obtained by using the coordinate distribution of the particles’ pixels, which is the position coordinate of the center of the particle, as shown below.

(5) $${\left(x,y\right)}_{particle}=\frac{{∯}_{particle}{\left(x_{i},y_{i}\right)}_{particle}dS_{i}}{S_{particle}}$$

In the Formula (5), ${\left(x,y\right)}_{particle}$ is the position coordinate of the particle; ${\left(x_{i},y_{i}\right)}_{particle}$ is the *i*th pixel’s coordinate in the particle; $dS_{i}$ is the area of the *i*th pixel, which equals 1 here; and, $S_{particle}$ is the area of the whole particle, which equals the amount of the pixels that are included in the particle. 

**Figure 8:**
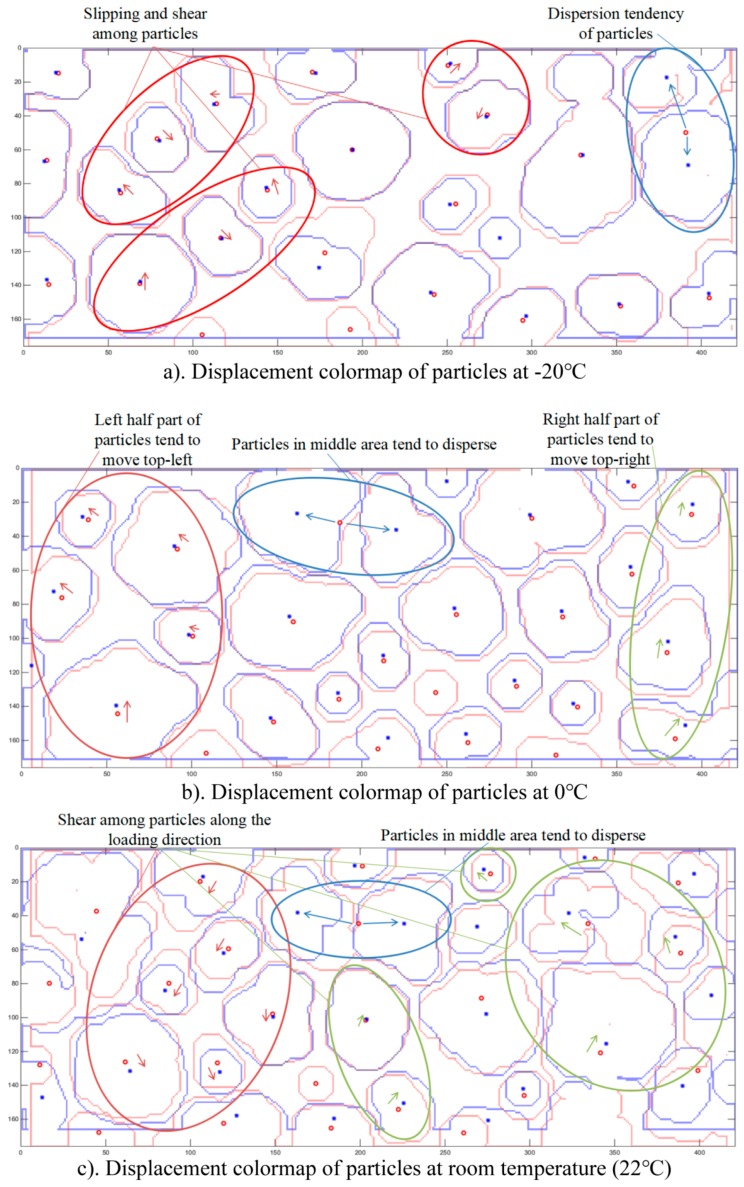

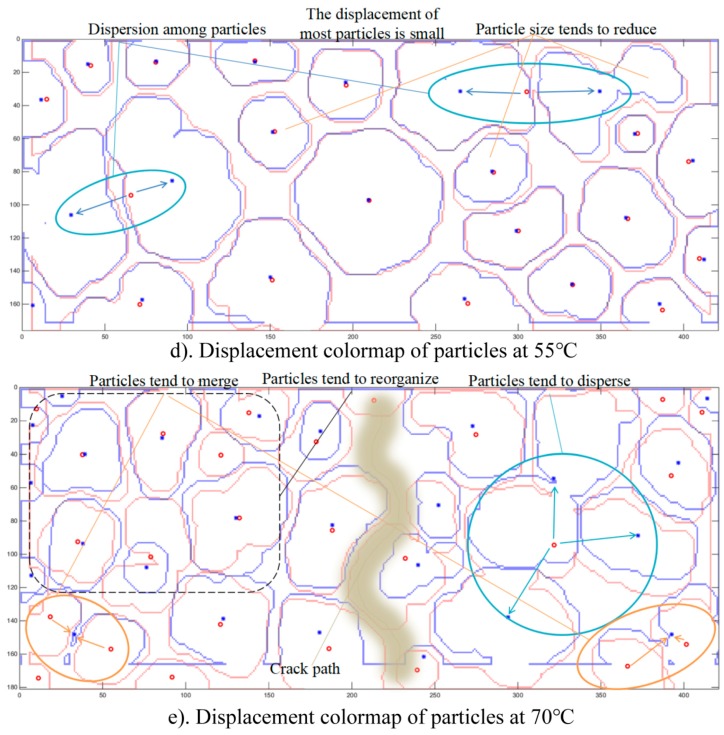
Displacement field of particles at (**a**) −20 °C; (**b**) 0 °C; (**c**) 22 °C; (**d**) 55 °C; and (**e**) 70 °C, respectively. The red ones and red circle markers show the original positions of particles, while the blue ones and blue star markers show the positions of particles after loading.

Because of the little loading displacement and the advantage of in-situ experiment, the thickness and the position of the specimen barely changed after loading. As a result, the particular middle slice can be accurately identified and located, and the particles barely moved in the thickness position of the specimen. When comparing the particle distribution morphology before and after loading, the interior displacement field of PBX at different temperatures can be obtained, as shown in Figure 8. The red ones and red circle markers show the original positions of particles, while the blue ones and blue star markers show the positions of particles after loading.

Through the above analysis, it can be concluded that: 1. at −20 °C to room temperature, which are all under the glass transition temperature of the binders, there are slipping and shear among the particles. Dispersion of particles can also be seen, which is proved as particle break by SEM detection; 2. at 0 °C, it can be observed that left half of particles move top-left, while right half of particles move top-right, which is exactly same as the test loading method, the top loading head is stable while the bottom loading head move upwards. This implies that the micro-structure evolution is the same as the macro-mechanical behavior; 3. at room temperature, shear along the loading direction is observed, which is because the limitation and the slight unbalance of the loading heads; 4. particles in the middle part of the specimen tend to disperse, which agrees with the fracture behavior under Brazilian test loading method; 5. when the temperature is up to 55 °C, which is above the glass transition temperature of binders, the binders begin to transform from glass state to high elastic state, so the binders soften. Therefore, the binder’s module reduces while the viscosity enhances, and it has better liquidity. So, the displacement of most particles is smaller, while the particles tend to disperse rather than displacing. The volume of binders tends to expending, accordingly, it can be seen that the particle size tends to reduce; 6. when temperature is higher and up to 70 °C, the binders are totally on high elastic state, the viscosity and the liquidity are more significant, resulting in a greater tendency of binder expending and particle reorganization. Particles tend to reshape rather than displacing, and there is complex structure evolution inside the specimen; and, 7. when comparing with the SEM images, it can be concluded that at lower temperatures, the dispersion of particles is mainly the break of particles, while at higher temperatures, is mainly debondings and break of binders.

## 5. Conclusions

A series of Brazilian tests of TATB-PBX at different temperatures were conducted, while the evolution of micro-structure inside the specimens was obtained through in-situ μCT observation. Through the digital imaging process, CT slice images were analyzed. Fracture degree and complexity were defined and were used to quantitatively describe the fracture characteristics at different temperatures. The fractal characteristic of cracks was also analyzed by using box-counting method. The interior displacement fields of particles were also studied and all of the above results were also proved by SEM detection. 

The 3D crack morphology was obtained, and it could be seen clearly and directly at lower temperatures that the cracks were straighter while at higher temperatures, especially above the glass transition temperature of binders, the cracks were more tortuous. The fracture degree was used to characterize the level of the fractures; while the fracture complexity was used to characterize the tortuosity character of the fractures. It could be seen that the fracture complexity tended to increase as the temperature rose, which implied a more tortuous crack. The fractal dimension of cracks showed a great minus linear relation with the temperature, which meant that as the temperature rose, the fracture fractal dimension decreased and the fracture surface was smoother. The interior displacement fields of particles showed the micro-structure evolution inside the specimens, which agreed with the macro-mechanical behavior at different temperatures. In SEM images, the typical morphology of fractures at different temperatures was obtained and proved the above conclusions. It was found that at lower temperatures, especially lower than the glass transition temperature of binders, there was slipping and shear among particles, and particles tended to displace and disperse; while at higher temperatures, especially above the glass transition temperature of binders, there was reorganization of particles and particles tended to merge, disperse, and reduce sizes, rather than displacing.
